# ATDMNet: Multi-Head Agent Attention and Top-k Dynamic Mask for Camouflaged Object Detection

**DOI:** 10.3390/s25103001

**Published:** 2025-05-09

**Authors:** Rui Fu, Yuehui Li, Chih-Cheng Chen, Yile Duan, Pengjian Yao, Kaixin Zhou

**Affiliations:** 1 Faculty of Computer and Software Engineering, Xihua University, Chengdu 610097, China; ruifu@stu.xhu.edu.cn (R.F.); 3120220912210@stu.xhu.edu.cn (P.Y.); 2 Engineering Research Center for ICH Digitalization and Multi-Source Information Fusion, Fujian Polytechnic Normal University, Fuzhou 350300, China; 3 Department of Automatic Control Engineering, Feng Chia University, Taichung 40724, Taiwan, China; 4 Faculty of Information and Software Engineering, University of Electronic Science and Technology of China, Chengdu 611731, China; 2023090906011@std.uestc.edu.cn; 5 Faculty of Management, Xihua University, Chengdu 610097, China; kaixinzhou@stu.xhu.edu.cn

**Keywords:** camouflaged object detection, self-attention network, feature extraction, digital images, visualization

## Abstract

Camouflaged object detection (COD) encounters substantial difficulties owing to the visual resemblance between targets and their environments, together with discrepancies in multiscale representation of features. Current methodologies confront obstacles with feature distraction, modeling far-reaching dependencies, fusing multiple-scale details, and extracting boundary specifics. Consequently, we propose ATDMNet, an amalgamated architecture combining CNN and transformer within a numerous phases feature extraction framework. ATDMNet employs Res2Net as the foundational encoder and incorporates two essential components: multi-head agent attention (MHA) and top-k dynamic mask (TDM). MHA improves local feature sensitivity and long-range dependency modeling by incorporating agent nodes and positional biases, whereas TDM boosts attention with top-k operations and multiscale dynamic methods. The decoding phase utilizes bilinear upsampling and sophisticated semantic guidance to enhance low-level characteristics, hence ensuring precise segmentation. Enhanced performance is achieved by deep supervision and a hybrid loss function. Experiments applying COD datasets (NC4K, COD10K, CAMO) demonstrate that ATDMNet establishes a new benchmark in both precision and efficiency.

## 1. Introduction

Camouflaged object detection (COD) [1] is a persistent and challenging domain within computer vision, concentrating on the identification of objects purposefully manufactured to coordinate with sophisticated scenes. These targets tend to be concealed leveraging innovative techniques, including alterations in color, form, texture, or illumination, complicating their identification via conventional visual inspection systems. Accurately identifying such targets becomes crucial for various practical applications, including military reconnaissance for target recognition, concealed animal monitoring in wildlife protection, and the detection of illegal intrusions in security surveillance systems, among others.

**Figure 1:**
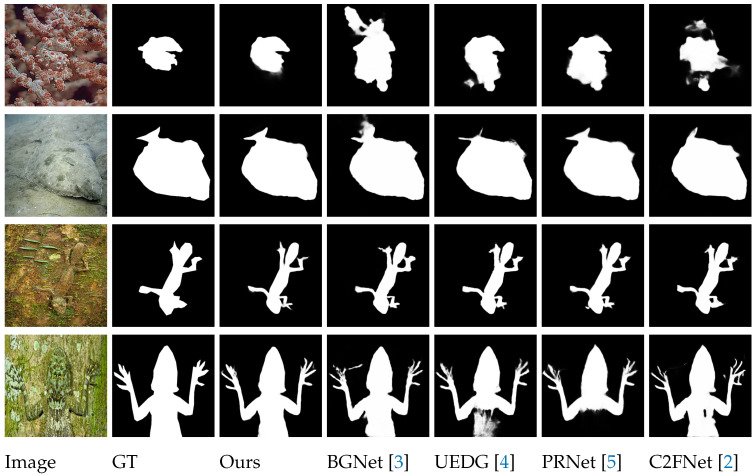
A visual comparison of our ATDMNet with contemporary SOTA methods [2,3,4,5], such as BGNet [3] and PRNet [5], for COD. Certain approaches inadequately segment the region, failing to reliably gather and forecast tracked items.

Camouflaged objects typically exhibit complicated feature spaces and multiscale information, hampering the ability of existing methods to effectively and comprehensively extract and represent these characteristics, thus restricting detection accuracy. Notwithstanding breakthroughs in CNN-based COD, intrinsic constraints endure the following: (1) constrained receptive field for global context: the confined character of convolutional procedures limits the modeling of long-range dependencies; (2) inappropriate multiscale feature fusion: unsophisticated techniques frequently produce redundant or conflicting feature interactions; (3) boundary ambiguity: recurrent pooling operations decline spatial resolution, compromising the precision of edge delineation. Even SOTA methods [2] are insufficient for achieving the accurate segmentation of camouflaged targets in complex scenarios. The challenge illustrated in Figure 1 mostly evolves from the model’s deficient selectivity in processing scale-related feature signals, impeding the distinction between camouflaged objects and closely comparable backgrounds. Therefore, mitigating this issue involves the enhanced usage of multiscale feature fusion and local feature attention.

To take on these problems, we render an updated camouflaged target detection approach, ATDMNet, that boosts detection performance while preserving computational economy. ATDMNet attains SOTA consequences at the site, surpassing rival methodologies in both parameter efficiency and computational efficacy. ATDMNet’s network architecture leverages an end-to-end design structured into three primary stages: feature extraction, information integration, and guided refinement.

During the feature extraction phase, ATDMNet utilizes Res2Net as the foundational encoder. The input image undergoes processing via convolutional layers, batch normalization, activation functions, and max-pooling layers, facilitating the extraction of multi-level semantic features with residual block layers. The features are further refined by adopting GFM layers, merging the superficial representation strengths of convolutional neural networks (CNNs) with the profound semantic modeling skills of transformers. This hybrid method markedly raises the model’s target localization effectiveness.

During the information integration phase, the multi-head agent (MHA) mechanism is employed to facilitate a bidirectional attention mechanism with agent nodes and positional biases. The top-k dynamic mask (TDM) module is concurrently utilized to create a multi-tiered attention mechanism. This approach utilizes convolution to offer query, key, and value representations, successfully incorporating multiscale global information and augmenting the model’s ability to represent and integrate characteristics.

During the guided refinement phase, the decoder incrementally reconstructs the feature map dimensions via an attention mechanism in conjunction with bilinear upsampling. This approach generates an individual channel output, exploiting high-level semantic information to enhance low-level features, which is employed to further augment the feature refinement process. A deep supervision technique and a hybrid loss function are employed to separately direct the learning process at each level of the decoder, markedly boosting the model’s identification and generalization skills. Collectively, these innovations empower ATDMNet to achieve the precise identification and segmentation of disguised targets, establishing an entirely novel norm in the domain.

To investigate the viability of the proposed ATDMNet methodology, we execute extensive and thorough experiments on three prominent COD benchmark datasets, including NC4K [6], COD10K [7], and CAMO [8]. The experimental findings indicate a weighted F-measure of **0.804** and a mean absolute error (MAE) of **0.021**, surpassing the second-best model, PRNet [5], which attains values of 0.799 and 0.023, respectively, on the COD10K-test dataset. ATDMNet reaches new SOTA performances across all benchmarks. This study’s principal contributions are as follows:

(1) We present a unique ATDMNet architecture that effectively blends feature information across several scales, showcasing improved adaptability to the feature properties of disguised objects at various resolutions. This capacity substantially improves overall detection efficacy, facilitating more precise identification in intricate situations.

(2) The multi-head agent (MHA) module and the top-k dynamic mask (TDM) module are implemented to improve the model’s responsiveness to local features. These modules proficiently capture long-range connections and enhance the dissemination of information globally, hence strengthening the model’s ability to detect disguised items in various contexts.

(3) Through comprehensive quantitative and qualitative testing, we confirm that ATDMNet exemplifies remarkable proficiency in modeling scale-related information and capturing border aspects. The findings suggest that ATDMNet attains exceptional accuracy and reliability in disguised target detection, establishing an unprecedented standard in the domain.

## 2. Related Work

### 2.1. Camouflaged Object Detection

Conventional COD methodologies [9] typically rely on saliency detection and segmentation algorithms to identify distinctive characteristics. Nevertheless, in intricate camouflage situations, these techniques frequently exhibit suboptimal performance owing to their restricted ability to precisely differentiate the specific distinctions between concealed items and their ambient background. Consequently, typical techniques sometimes necessitate the incorporation of supplementary features or auxiliary data to augment detection precision and reinforce their resilience in more discerning settings.

In recent years, convolutional neural network (CNN)-based strategies for COD have substantially enhanced detection accuracy. These methodologies can be classified into three primary strategies: (a) boundary guidance: BGNet [3] investigates supplementary edge semantic information pertinent to objects and delineates prominent structural characteristics of the target. BSA-Net [10] utilizes a dual-stream separation attention module to distinguish between foreground and background, integrating this with a boundary guidance module to augment boundary feature comprehension, thus enhancing detection efficacy. (b) Frequency domain information enhancement: Zhong et al. [11] incorporated frequency domain information into CNNs by employing a multi-head self-attention mechanism and high-order relationship modules to retrieve and incorporate cross-domain features, thereby augmenting the model’s capacity to detect subtle camouflage patterns. FSEL [12] introduces an entanglement transformer block (ETB) and a dual-domain inverse resolver, adeptly capturing correlations across several frequency bands and synthesizing cross-domain features for enhanced detection. FPNet [13] employs a dual-stage frequency-range-sensing technique that enhances detection efficacy via the coarse and fine localization of camouflaged targets. FGSA-Net [14] transfers features into the frequency domain and dynamically modulates the intensity of frequency components by organizing and interacting with them within non-overlapping circular sections of the spectrogram. (c) Utilization of contextual information: Spider [15], a cohesive parameter-sharing model, evolves and differentiates robust context dependence (CD) across multiple fields, combining one-shot training and image-mask group prompts to strengthen the model’s efficacy. C2F-Net [2] applies cross-layer context-aware features, constructs a cascaded attention-driven cross-layer fusion module (ACFM) and a dual-branch global context module (DGCM), and computes attention coefficients for multi-level features to enhance and amalgamate salient features.

### 2.2. Transformers in Computer Vision

Since the introduction of the transformer architecture, it has swiftly attained transformative breakthroughs in the domain of natural language processing (NLP) [16]. Recently, the transformer architecture has been customized for implementation in computer vision. In contrast to typical CNNs, transformers utilize the self-attention technique to comprehensively capture dependencies among input data. This attribute renders it appropriate for diverse visual domains. It has been extensively applied in tasks, encompassing critical domains such as image classification, semantic segmentation, object detection, and salient object detection.

Transformer-based models are progressively emerging as a predominant trend in the COD domain. HGINet [17] employs the Hierarchical Graph Interactive Transformer (HGIT) for facilitating bidirectional communication across hierarchical features. FSPNet [18] adopts non-local techniques to augment the local representation of transformers and explores the interaction of adjacent tags through high-order relationships grounded in graphs. CamoFormer [19] presents the Simple Masked Separable Attention (MSA) mechanism, which segments multi-head self-attention into three components, each employing distinct masking methods. UGTR [20] amalgamates probability with the transformer architecture and embraces uncertainty to facilitate reasoning in ambiguous situations.

Our ATDMNet is constructed upon the prevailing transformer structure. Rather than emphasizing on the creation of an entirely new architecture, our objective is to investigate alternative approaches to maximize the efficacy of self-attention mechanisms in COD tasks. We incorporate multi-level semantic characteristics into the multi-head agent attention modules, successfully emphasizing features at various scales through the integration of top-k operations and multiscale attention masks. This architecture renders our study distinctly unique among transformer-based COD methodologies.

## 3. Proposed ATDMNet

### 3.1. Overall Architecture

**Figure 2:**
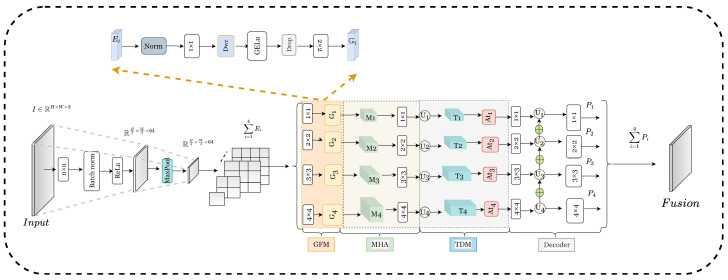
ATDMNet addresses challenges in binary segmentation. The GFM and MHA modules integrate multi-level features retrieved by Res2Net across many scales within the encoder. Top-k dynamic mask (TDM) is employed to enhance feature representation. The decoder derives the single-channel output using upsampling and convolutional layers to reconstruct the feature map. The structures of MHA and TDM are presented in Figure 3 and Figure 4.

We utilize an encoder–decoder architecture to construct our ATDMNet. ATDMNet constitutes a comprehensive training framework, as seen in Figure 2.

**Encoder:** We rely on Res2Net as the encoder by default, as the visual transformer has demonstrated strong effectiveness in binary segmentation tasks. The input image $I\in {ℜ}^{H\times W\times 3}$ is processed through many n×n convolutional layers, batch normalization, and activation functions, resulting in the output ${ℜ}^{H/2\times W/2\times 64}$. Subsequently, a max-pooling layer is applied to downsample the feature map by fifty percent, resulting in the output ${ℜ}^{H/4\times W/4\times 64}$. This is succeeded by four essential residual blocks, each generating its corresponding output $\left\{f_{1}^{\frac{H}{4}\times \frac{W}{4}\times 64},f_{2}^{\frac{H}{8}\times \frac{W}{8}\times 128},f_{3}^{\frac{H}{16}\times \frac{W}{16}\times 256},f_{4}^{\frac{H}{32}\times \frac{W}{32}\times 512}\;\right\}$.

(1) $$\begin{array}{c} E_{i}=Maxpool\cdot \left(ReLu\cdot \left(Norm\cdot conv_{n\times n}\left(Input\right)\right)\right) \end{array}$$

We feed the multi-level semantic features ${\left\{E_{i}\right\}}_{i=1}^{4}$ from the encoder into the GFM. The GFM model is utilized to achieve an improved equilibrium between efficiency and performance by executing n×n convolutions and feature transformation:

(2) $$\begin{array}{cc} G_{i} & =Dropout(E_{i}\cdot Conv_{1\times 1}\cdot Dwc\cdot GELu\cdot Conv_{2\times 2}) \end{array}$$

The features obtained by GFM transformation are transferred to MHA together for multiscale feature fusion to generate ${\left\{M_{i=1}^{4}\right\}}^{f}$. The process is defined as follows:

(3) $${\left\{M_{i=1}^{4}\right\}}^{f}=Conv_{n\times n}\left(MHA\left({\left\{G_{i}\right\}}_{i=1}^{4}\right)\right)$$

Subsequently, within the context of the preceding feature fusion, varied attention masks are applied to spotlight the elements of diverse scales highlighted by the TDM module, hence augmenting the model’s representational capacity and generalization efficacy. This procedure is delineated as follows:

(4) $${\left\{T_{i=1}^{4}\right\}}^{f}=TDM\left(F_{up}{\left\{M_{i=1}^{4}\right\}}^{f}\right)$$

The acquired features are input into the decoder to produce a feature fusion map $P_{fus}\in R^{1\times H\times W}$.

### 3.2. Agent Top-k Dynamic Mask (ATDM)

Acquiring scale information across numerous characteristic areas is essential for the detection of camouflaged objects. Consequently, augmenting feature representation and emphasizing local features are essential for enhanced detection accuracy. Consequently, we present an innovative method that integrates the conventional multi-head self-attention mechanism with agent tokens. We present Agent Top-k Selection and Dynamic Masking, which enhance the model’s capacity to deal with long-range dependencies while preserving sensitivity to intricate local details.

In our methodology, distinct attention heads are assigned specific responsibilities to enhance the model’s capability to concentrate on significant features. The implementation of agent features is crucial in this process. Utilizing adaptive average pooling, we derive crucial information from the input feature map, which is subsequently employed to generate various attention matrices. The Top-k maximum values are chosen to enhance the attention mechanism, hence augmenting the model’s capacity to identify pertinent characteristics. This approach boosts feature attention while simultaneously decreasing computing complexity, thus promoting the more precise and efficient recognition of camouflaged objects.

Our ATDM is constructed on a multi-head self-attention fusion agent to minimize computation, specifically multi-head self-attention (MHSA). Let $X\in R^{H\times W\times C}$, where *H* and *W* signify height and width, respectively, and *C* denotes the number of channels. The equation for MHSA is as follows:

(5) $$A\left(Q,K,V\right)=\mathrm{Softmax}\left(\frac{QK^{T}}{\sqrt{d_{k}}}\right)\cdot V$$

In this context, *Q* represents the query matrix, *K* denotes the key matrix, *V* signifies the value matrix, and $d_{k}$ refers to the dimension of the key vector, which is utilized to scale the dot product for the purpose of stabilizing the Softmax function. These calculations are executed, making use of parallel attention mechanisms.

(6) $$H_{i}=A\left(QW_{i}^{Q},KW_{i}^{K},VW_{i}^{V}\right)$$

Among these, $W_{i}^{Q},W_{i}^{K},$ and $W_{i}^{V}$ are the transformation matrices for the query, key, and value of the *i*-th head, respectively. The results from all heads are concatenated and subsequently subjected to a final linear transformation, followed by layer normalization, which generates the output of the multi-head attention.

(7) $$MHSA\left(Q,K,V\right)=Concat\left(H_{1},H_{2},\ldots ,H_{h}\right)W^{O}$$

The aforementioned MHSA facilitates the concurrent operation of several independent attention heads, each focusing on distinct facets of the incoming data. The attention mechanism is utilized to encode spatial information, allowing the model to effectively capture interdependence across spatial dimensions.

#### 3.2.1. Multi-Head Agent (MHA)

The primary distinction in our MHSA is the incorporation of agent nodes (agent tokens) and the position and agent bias for multiscale feature integration and sensitivity to spatial location. To accomplish this, we transform the input tensor *X* from the shape (B,C,H,W) to (B,N,C), where N=H×W represents its flattened sequence form.

(8) $$\begin{array}{cc} X & =Reshape(B,H\times W,C)\\ X & \in R^{B\times H\times W\times C}\to X\in R^{B\times N\times C} \end{array}$$

If $Sr_{ratio}>1$, spatial reduction (SR) is executed first. The size of the convolution kernel is the $sr_{ratio}$. Setting $stride=sr_{ratio}$ results in the downsampling of the spatial dimensions of the input feature map. The particular operation is as follows:

(9) $$\begin{array}{c} X_{sr}=Conv2D\left(X,kernel_{size}=sr_{ratio},stride=sr_{ratio}\right)\;\;\;\in R^{B\times C\times \frac{H}{sr}\times \frac{W}{sr}} \end{array}$$

Subsequently, compute the key value, where $\frac{N}{sr}=\left(\frac{H}{sr}\right)\times \left(\frac{W}{sr}\right)$. When $Sr_{ratio}>1$, we perform spatial downsampling as follows:

(10) $$\begin{array}{cc} \left[K,V\right] & =G_{i}\cdot Reshape\cdot X_{sr}\cdot Norm\cdot Reshape\left(B,-1,-2,C\right)\;\;\;\in R^{B\times \frac{N}{sr}\times 2C}\\ else\\ \left[K,V\right] & =G_{i}\cdot Reshape\left(B,-1,2,C\right) \end{array}$$

Ultimately, partitioning into K and V, compute the query Q and then employ adaptive average pooling from Q to generate tokens.

(11) $$\begin{array}{cc} K,V & =[K,V]\cdot Depart(0,1)\\ Q & =G_{i}\cdot reshape\left(B,N,C\right)\\ Tokens & =Reshape\cdot AvgPool(Q) \end{array}$$

**Figure 3:**
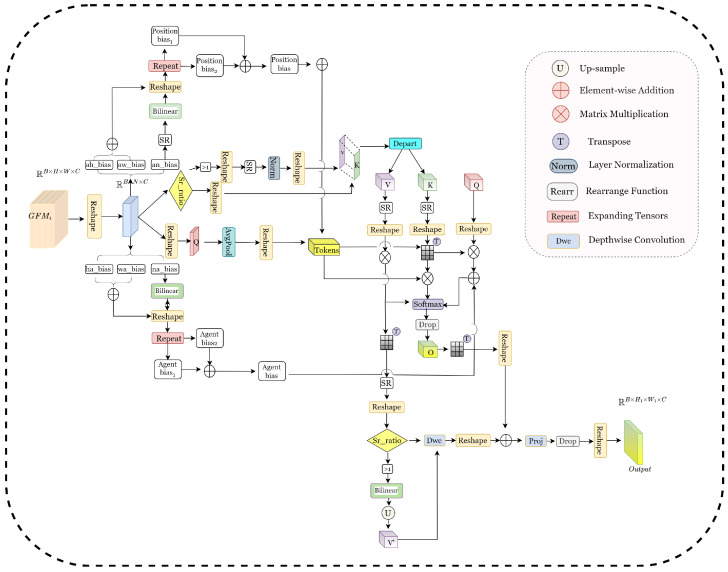
Multi-head agent (MHA).

Figure 3 illustrates that in MHA, position bias and agent bias are two fundamental position-aware biases that establish bidirectional attention and simultaneously capture both global and local information. They are utilized for agent-to-grid attention and grid-to-agent attention, respectively. The model can explicitly encode relative position information through position bias and agent bias. However, their mechanisms of action are fundamentally different.

From the perspective of structural design and effect, position bias is employed to assess the attention of agent tokens towards the spatial grid, which comprises three components: (1) $an_{bias}$ ($B_{an}$): the spatial correlation between tokens and their immediate vicinity; (2) $ah_{bias}$ ($B_{ah}$): the inclination of tokens towards the horizontal orientation; (3) $aw_{bias}$ ($B_{aw}$): the inclination of tokens towards the vertical orientation. Upon the initialization of $B_{an}$, sampling from a truncated normal distribution is employed. From this, we can deduce that the positional bias is as follows:

(12) $$B_{an}\to X_{sr}\cdot Bilinear$$

During the computation of attention, $B_{an}$ is adjusted in size according to $sr_{ratio}$ to correspond with the spatial dimensions of K.

(13) $$\begin{array}{cc} Pos_{bias1} & =B_{an}\cdot Reshape\cdot Repeat\\ Pos_{bias2} & =(B_{ah}+B_{aw})\cdot Reshape\cdot Repeat\\ Pos_{bias} & =Pos_{bias1}+Pos_{bias2} \end{array}$$

Agent bias is employed in the attention computation of spatial grids to tokens and comprises three components: (1) $na_{bias}$ ($B_{na}$): modeling the local neighborhood association of spatial position to tokens; (2) $ha_{bias}$ ($B_{ha}$): modeling the preference of the horizontal position to tokens; (3) $wa_{bias}$ ($B_{wa}$): modeling the preference of the vertical position to tokens. The initialization procedure for $B_{na}$ mirrors that of $B_{an}$, but the dimensions are modified to (H,W) to correspond with the input resolution.

(14) $$\begin{array}{cc} Agent_{bias1} & =B_{na}\cdot Bilinear\cdot Reshape\cdot Repeat\\ Agent_{bias2} & =(B_{ha}+B_{wa})\cdot Reshape\cdot Repeat\\ Agent_{bias} & =Agent_{bias1}+Agent_{bias2} \end{array}$$

Regarding functional objectives, $Pos_{bias}$ serves as a dynamic receptive field controller that modulates the agent’s attention distribution range via the temperature coefficient; $Agent_{bias}$ functions as an information routing selector, utilizing learnable parameters to establish the information aggregation pathway from various spatial locations to the agent token, leading to a distributed feature integration akin to a voting mechanism.

Meanwhile, several positional biases are implemented to modify the attention weights. These biases establish supplementary connections between agent nodes and ordinary nodes, enabling the recording of more intricate interaction patterns and upgrading the capacity of the model to learn spatial dependencies.

For agent-to-grid attention,

(15) $$\begin{array}{cc} Attn_{a\to g} & =Dropout\left(softmax\left(\frac{Tokens\times {\left(K\cdot X_{sr}\right)}^{T}}{\sqrt{d_{k}}}+Pos_{bias}\right)\right)\\ O_{a\to g} & =Attn_{a\to g}⊗V \end{array}$$

For grid-to-agent attention,

(16) $$\begin{array}{cc} Attn_{g\to a} & =Dropout\left(softmax\left(\frac{Tokens^{T}\times Q}{\sqrt{d_{k}}}+Agent_{bias}\right)\right)\\ O_{g\to a} & =Attn_{g\to a}⊗O_{a\to g} \end{array}$$

Among them, $Pos_{bias}$ and $Agent_{bias}$ represent the positional biases from the agent to the grid and from the grid to the agent, respectively.

The output is generated. Initially, the dimensions of $O_{g\to a}$ are modified. If $Sr_{ratio}>1$, carry out bilinear interpolation and upsampling on V and incorporate deep convolution into V to augment local features. Ultimately, execute linear projection. MHA enhances the model’s sensitivity to local features through the integration of agent nodes and promotes efficient information flow using a bidirectional attention mechanism.

(17) $$\begin{array}{cc} O & =O_{g\to a}^{T}\cdot Reshape\\ Output & =Dropout\left(Proj\left(O+\left(DWc\left(V\right)\cdot Reshape\right)\right)\right) \end{array}$$

#### 3.2.2. Top-k Dynamic Mask (TDM)

This module implements a customized multi-head self-attention mechanism to capture spatial and channel dependencies in feature maps, employing qkv and Dwc layers to produce queries for the input features via convolution operations. Specifically, *Q* denotes the query, but *K* and *V* signify distinct representations of the input features. Meanwhile, transform into multi-head format using the Rearrange function to execute attention computations across various heads.

(18) $$\begin{array}{cc} QKV & =Conv_{1\times 1}\cdot X\;\;\;\;\in R^{B\times H_{1}\times W_{1}\times 3C}\\ QKV_{dw} & =Dwc\left(QKV\right),\;Dwc\in R^{3C133}\\ Q,K,V & =Rearr\left(QKV_{dw}\right)\in R^{B\times H_{1}\times W_{1}\times C} \end{array}$$

Afterwards, normalize *K* and *V* along the final dimension.

(19) $$\begin{array}{c} Q=\frac{Q}{{\left||Q|\right|}_{2}},K=\frac{K}{{\left||K|\right|}_{2}} \end{array}$$

After that, calculate the original attention matrix, where $d_{k}=\frac{C}{head}$ serves as the scaling factor.

(20) $$Attn=\frac{\left(Q\cdot K^{T}\right)}{\sqrt{d_{k}}}⊗Temp$$

$Temp\in R^{N_{heads}\times 1\times 1}$ is a trainable temperature parameter utilized to modulate the attention scores, enabling the model to adaptively regulate the focus intensity for each head throughout training. This module creates an initial attention matrix (Attn) by assessing the similarity between *Q* and *K*, thereafter selecting a specified number of components based on the attention scores through the top-k operation. Attention masks of varying scales ($Mask_{1}$ to $Mask_{4}$) regulate the allocation of attention across many levels, progressively augmenting the quantity of selected attention elements to establish a multi-tiered attention mechanism.

(21) $$\begin{array}{cc} Index_{i} & =Top_{k}\left(Attn,k\right),\\ Mask_{i} & =Scatter\left(Mask_{i},-1,{\mathrm{index}}_{i},1.\right)\\ Attn_{i} & =Where\left(Mask_{i}>0,Attn,-\right)\\ k & =\left(\frac{C}{2},\frac{2C}{3},\frac{3C}{4},\frac{4C}{5}\right),i=\left(1,2,\cdot \cdot \cdot ,4\right) \end{array}$$

Upon implementing Softmax regularization at each attention level ($Attn_{1}$, $Attn_{2}$, etc.), the resultant attention matrices are multiplied by the value *V* to produce weighted outputs ($o_{1}$ to $o_{2}$).

(22) $$output_{k}=Softmax\left(Attn_{i}\right)\times V$$

These outputs correspondingly delineate the attention ranges of varying densities. For instance, $Attn_{1}$ exclusively accounts for the top 50% of attention values, while $Attn_{4}$ encompasses 80%.

(23) $$Output=\sum_{k=1}^{4}\lambda_{k}\times Output_{k},\;\lambda_{k}=Atten_{i}$$

In the concluding phase, the outputs from the several attention layers are weighted and consolidated, utilizing weighting parameters $Attn_{1}$ to $Attn_{4}$. Thereafter, the consolidated output undergoes a projection and transformation procedure to produce the integrated multiscale attention output.

(24) $$\begin{array}{cc} Output & =Rearr(Output,B,C,H_{1},W_{2})\\ F_{output} & =W_{proj}*Output\;\;\;\;\;\;\left(W_{proj}\in R^{C\times C\times 1\times 1}\right) \end{array}$$

**Decoder:** Considering $C_{out}$ as the number of output channels, the shape of the transformed feature map is $R_{i}^{N,C_{out},H_{n},W_{n}}$, where *N* signifies the batch size, $C_{out}$ indicates the output channels, and $H_{n}$ and $W_{n}$ indicate the height and width of the feature map at scale *n*. Refer to Figure 2 for specifics. The multiscale fusion feature map is derived by the attention mechanism and upsampling process, and it can be represented as follows:

(25) $$\begin{array}{cc} o_{4} & =At_{4}\left(o_{4}\right)\\ o_{3} & =At_{3}\left(T_{3}+F_{up}\left(o_{4}\right)\right)\\ o_{2} & =At_{2}\left(T_{2}+F_{up}\left(o_{3}\right)\right)\\ o_{1} & =At_{1}\left(T_{1}+F_{up}\left(o_{2}\right)\right) \end{array}$$

$F_{up}$ denotes the bilinear upsampling operation employed for aligning spatial dimensions. Thereafter, the feature map undergoes processing via a 1×1 convolutional layer to yield a single-channel output, which can be formally articulated as follows:

(26) $$\begin{array}{cc} P_{i} & =Conv_{2d}\left(o_{i}\right)\;\;\left\{i=1,2,\ldots ,4\right\}\\ Fusion & =\sum_{i=1}^{4}P_{i} \end{array}$$

### 3.3. Loss Function

In accordance with [21], we refer to the predictions generated by the ATDMNet decoder as ${\left\{P_{i}\right\}}_{i=1}^{4}$. All predicted maps $P_{i}$, with the exception of the final prediction map $P_{1}$, are employed for multiscale fusion as previously delineated. In the phase of training, each $P_{i}$ is upsampled to correspond with the dimensions of ground truth Mask, and a weight matrix $w_{eit}$ is calculated to highlight the significance of boundary regions. All projections are governed by the BCE loss and IoU loss, with the final loss calculated as the average of the BCE and IoU losses. The overall loss function is the aggregate of multi-stage losses, each with a distinct weight.

(27) $$L\left(P,G\right)=\frac{\sum_{i=1}^{n}w_{eit}^{i}\left\{L_{bce}\left(P_{i},G\right)+L_{iou}\left(P_{i},G\right)\right\}}{N}$$

## 4. Experimental Results

### 4.1. Experiment Setup

**Implementation details:** We utilize the PyTorch 1.8+ library [22] to implement ATDMNet. A pretrained Res2Net [23] functions as the encoder, whereas PVTv2 [24] acts as the primary backbone. We additionally provide findings using alternate architectures, including the transformer-based Swin Transformer [25] and the CNN-based ResNet [26]. The utilized optimizer is Adam [27], utilizing an initial learning rate of $5\times 10^{-5}$ and a weight decay of $1\times 10^{-5}$ to mitigate overfitting. The learning rate schedule employs Cosine AnnealingLR, which is appropriate for small batch training in this experiment and mitigates local optimality through periodic restarts. The maximum period $T_{max}$ is 20, and the lowest learning rate is $1\times 10^{-5}$. Gradient clipping is employed for regularization with a threshold of 0.5 to avert gradient explosion. All training datasets are sized at 448 × 448 pixels. The computational load is regulated while preserving details, in accordance with the layer architecture of PVTv2. Constrained by GPU video memory, the model is subjected to end-to-end training for 60 epochs, necessitating approximately 7 h with a batch size of 8 on an NVIDIA RTX 4090 24 GB (NVIDIA Corporation, Santa Clara, CA, USA), thus optimizing video memory utilization and batch stability.

**Datasets:** We assess our approach using three prevalent COD benchmarks: CAMO [8], COD10K [7], and NC4K [6]. CAMO comprises 2500 photos, with an equal distribution of camouflaged objects and non-camouflaged objects. COD10K comprises 5066 camouflaged photos, 3000 background images, and 1934 non-camouflaged photographs. NC4K is an extensive dataset comprising 4121 test photos. In accordance with prior studies, we utilize 1000 images from CAMO and 3040 images from COD10K for training, reserving the remaining images for testing.

**Evaluation Metrics:** To assess the initial three tasks, we employ the subsequent metrics: The mean absolute error (MAE) adaptive F-measure ($F_{\beta }$) [28], mean E-measure ($E_{\phi }$) [29], and structure measure ($S_{\alpha }$) [30] are referenced in [31]. Reduced values of M and elevated values of $F_{\beta }$, $E_{\phi }$, and $S_{\alpha }$ signify superior performance.

### 4.2. Qualitative Evaluation

#### Visualization Predictions

**Figure 5:**
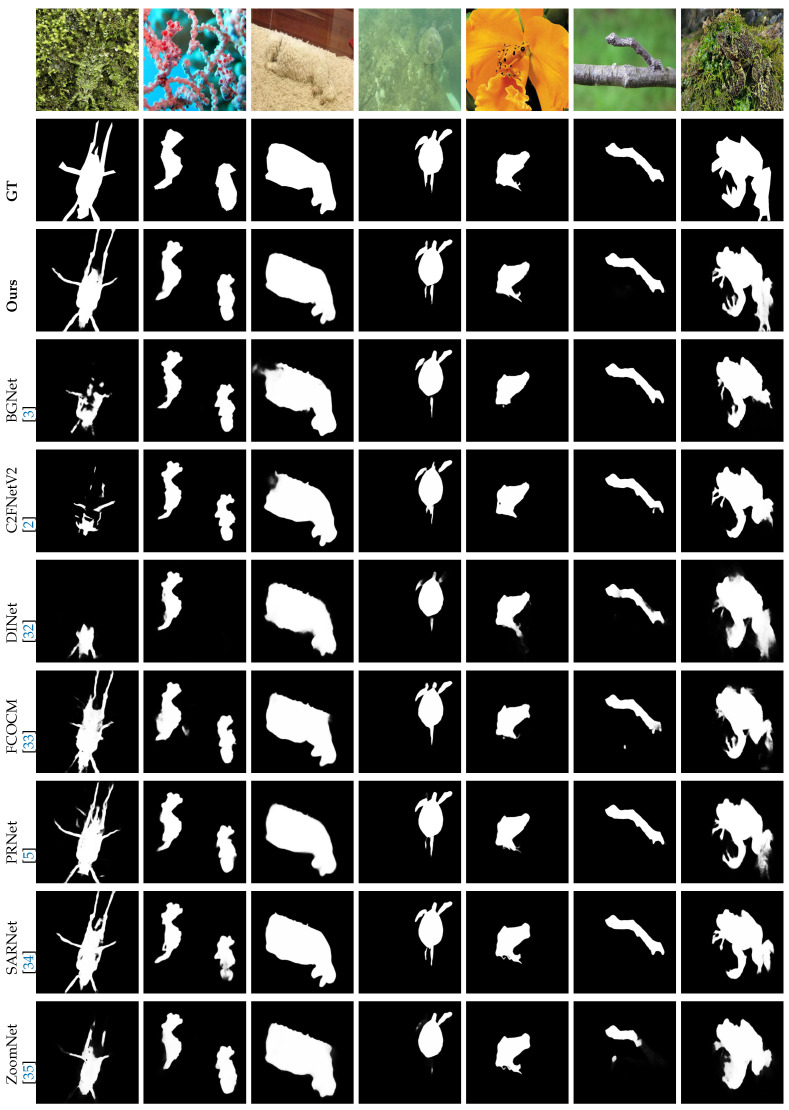
Visual comparisons of the predictions between the proposed ATDM and other SOTA methods (Baseline) [2,3,5,32,33,34,35]. Segmentation masks are shown in white.

Figure 5 presents a systematic comparison of the proposed ATDMNet against seven SOTA camouflaged object detection (COD) algorithms across several scenarios featuring multiple camouflaged items, ranging from Column 1 (Lizard) to Column 7 (Frog). Quantitative assessments indicate that current methodologies, such as BGNet [3], C2FNetV2 [2], and DINet [32], demonstrate considerable deficiencies in identifying low-contrast camouflaged objects. As an illustration, these approaches exhibit insufficient segmentation of the camouflaged lizard in Column 1, with significant false positives (e.g., erroneously categorizing background textures as feline characteristics). This boost in performance arises from ATDMNet’s hierarchical multiscale fusion design, which incorporates cross-scale attention methods to increase discriminative feature representation.

### 4.3. Quantitative Evaluation

#### 4.3.1. Comparison with Existing COD Methods

We contrast our ATDMNet with eight ResNet50-based SOTA COD models, including BGNet [3], BSANet [36], SegMaR [37], ZoomNet [35], C2FNet [2], FEDER [38], ICEG [39], and CamoFormer [19]; four Res2Net50-based models, including FDNet [40], SINetV2 [1], DINet [32], and FCOCM [33]; and seven transformer-based methods, including FPNet [41], FSPNet [42], SARNet [34], UEDG [4], VSCode [43], RISNet [44], and PRNet [5]. To ensure a fair comparison, the prediction results are either directly provided by the authors or generated using their well-trained models.

As observed in Table 1, our proposed ATDMNet consistently and markedly surpasses prior methods across all three benchmarks, without any requirement for any post-processing techniques or additional information for training. In comparison with contemporary CNN-based COD methodologies, including ZoomNet [35], FDNet [40], and SegMaR [37], which utilize multiple stages training and inference strategies that introduce additional computational burden, ATDMNet consistently attains significantly better outcomes across all benchmarks. Moreover, in comparison to transformer-based models (e.g., VSCode [43] and RISNet [44]), our approach surpasses them, establishing new SOTA benchmarks.

#### 4.3.2. Computational Efficiency Analysis

Table 2 illustrates that, regarding the correlation between parameter size and MACs, the Res2Net50 branch exhibits that ATDM-R2N (29.5 M/43.2 G) is markedly inferior to SINetV2 (51.1 M/68.5 G) and obtains the highest FPS (88.4). This is attributable to the multiscale residual architecture of Res2Net and the dynamic mask co-optimization of TDM, which eliminates superfluous feature computations. FDNet (28.7 M/45.6 G) demonstrates performance equivalent to ATDM-R2N, albeit with a marginally reduced FPS (85.6), suggesting that MHA’s proxy nodes enhance the efficacy of parallel computing.

We investigate the trade-off between FPS and computing efficiency, notably contrasting transformers and CNNs. Under identical MACs, FPS markedly exceeds that of CNN approaches (e.g., ATDM-R2N), implying that GPU parallel computing optimization for the transformer is more beneficial, particularly when the model minimizes superfluous attention computations (such as the top-k screening of TDM). Nonetheless, pure CNN technologies (such as BGNet and ZoomNet) are constrained by local receptive fields and necessitate additional layers to capture long-range dependencies, leading to an escalation in parameters and computational demands, yet yielding minimal innovations in speed.

### 4.4. Ablative Studies

To evaluate the sensitivity of local details in our proposed method, the capacity to incorporate global information, and the efficacy of each major module, we perform ablation studies on three datasets. We numerically and qualitatively illustrate the impact of local feature sensitivity and long-range dependence on the model.

#### 4.4.1. Validation of Module Combination

Table 3 demonstrates that the integration of MHA boosts $F_{\beta }$ from 0.742 to 0.769 (+2.7%), while MAE shrinks from 0.032 to 0.029. This improvement is attributed to MHA’s capacity to equilibrate the significance of global and local information, thereby alleviating redundant computations by dynamically modulating the attention range through spatial reduction ($X_{sr}$ in Formula (9)) and explicitly modeling spatial connections ($Pos_{bias}$ and $Agent_{bias}$ in Formulas (13) and (14)) with sparse interactions among agent nodes. With the integration of TDM, the $E_{\phi }$ for multiscale targets on NC4K increases by 0.9% (0.890→0.899); TDM obtains cross-scale complementarity with top-k screening and multi-level mask fusion. Simultaneously, in conjunction with channel standardization (Formula (19)) and the dynamic temperature parameter (Formula (20)), the attention strength is adaptively modulated to facilitate the implicit simulation of channel dependence. In COD10K, the combined application of MHA and TDM results in a 1.6% $F_{\beta }$ improvement (0.769→0.785), supporting the complementary optimization of both approaches via “feature enhancement” and “feature focusing”.

#### 4.4.2. Feature Visualization

**Figure 6:**
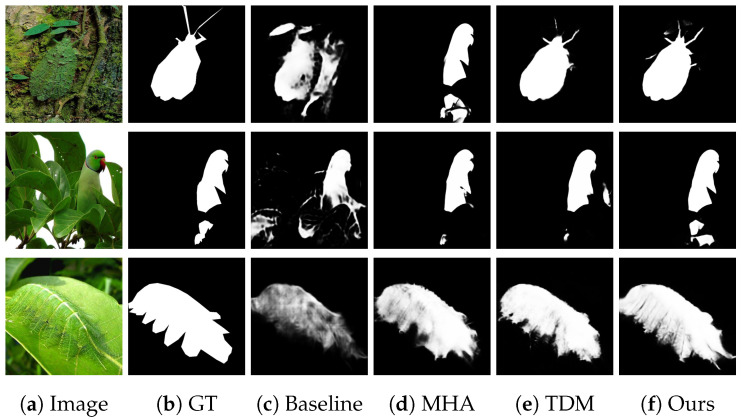
Results of the ablation study visualized. (**a**) Input image; (**b**) ground truth; (**c**) Baseline; (**d**) incorporating MHA; (**e**) incorporating TDM; (**f**) ATDMNet.

As illustrated in Figure 6d, the incorporation of MHA results in a transformation of the caterpillar limb outlines from indistinct (Baseline) to distinct, while simultaneously diminishing the false detection regions of analogous textures (e.g., leaf textures) in the background, attributable to the amplification of local features by the MHA’s agent nodes. As illustrated in Figure 6e, the incorporation of TDM transforms the parrot trunk area from fragmented (Baseline) to intact, while the interference response of the leaf backdrop diminishes (MAE falls by 0.002) due to TDM’s integration of multiscale characteristics via dynamic masks. As illustrated in Figure 6e, the comprehensive model facilitates the more precise segmentation of the transition zone between insect transparent wings and the backdrop, achieving a $E_{\phi }$ of 0.915.

#### 4.4.3. Balance Analysis of the Number of MHA Agent Nodes

We perform experiments utilizing MHA with differing quantities of agent nodes, establishing five experimental groups to train ATDMNet. The number of agent nodes is configured to various values (4, 8, 16, 32, and 64). The default top-k ratio of the fixed TDM module is 50%, and the number of dynamic mask levels is configured to three layers. As indicated in Table 4, with 16 agent nodes, the COD10K and CAMO datasets attain optimal values across several metrics, including $F_{\beta }$, M, $E_{\phi }$, and $S_{\alpha }$. In the NC4K dataset, the maximum $E_{\phi }$ value is recorded with eight agent nodes. Augmenting the number of agent nodes beyond 16 (e.g., to 32 or 64) results in performance deterioration, perhaps due to noise from duplicate calculations or overfitting.

#### 4.4.4. Adaptability Analysis of TDM Top-k Ratio

We established varying top-k ratios while maintaining a constant number of agent nodes in the MHA module and a fixed number of dynamic mask levels in the TDM module. According to Table 5, a 70% top-k ratio achieves the optimal performance across all three datasets. A low ratio results in the excessive filtration of essential features, causing reductions in $F_{\beta }$ and $E_{\phi }$. Conversely, an excessively high ratio introduces noise, resulting in reductions in MAE and $S_{\alpha }$. CAMO exhibits heightened sensitivity to the top-k ratio, demonstrating a substantial increase in $F_{\beta }$ at 70%. Consequently, for intricate situations, a top-k ratio of 70% to 80% is advised to achieve a balance between accuracy and robustness.

#### 4.4.5. Optimization Analysis of Dynamic Mask Level

**Figure 7:**
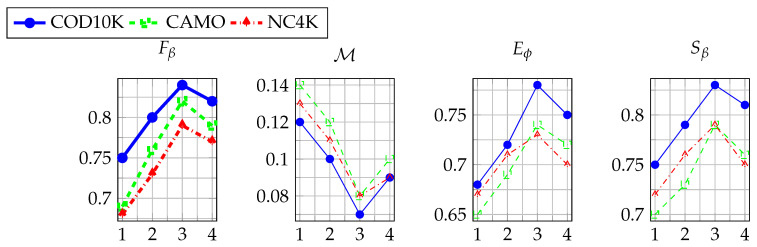
Influence of the number of dynamic mask levels on performance.

Expanding upon the sensitivity study of MHA and TDM hyperparameters, we further examine the influence of the number of dynamic mask layers on ATDMNet’s performance. The number of agent nodes is fixed at an optimal value of 16, and the top-k ratio is set at 70%. As depicted in Figure 7, a deficient number of layers results in the inadequate fusion of multiscale characteristics, leading to performance deterioration. Conversely, an overabundance of layers engenders computational redundancy, resulting in diminished performance (e.g., $F_{\beta }$ on COD10K declines from 0.84 to 0.82). Upon thorough investigation, we conclude that three layers represent the global optimum, attaining peak performances across all three datasets while efficiently incorporating multiscale features.

## 5. Conclusions

We present ATDMNet, a unique architecture for camouflaged object detection (COD) that combines a Res2Net-based encoder with a multi-dead agent attention (MHA) mechanism and a top-k dynamic mask (TDM) module to tackle issues in multiscale feature fusion and boundary refining. The MHA improves local sensitivity and global dependency modeling through agent nodes and positional biases, whilst the TDM emphasizes essential features via dynamic multiscale masking. The decoder utilizes bilinear upsampling and high-level semantic guidance to enhance low-level features, resulting in the accurate segmentation of disguised targets. ATDMNet’s resilience in intricate circumstances and computing efficacy position it as a standard for forthcoming COD research.

## Figures and Tables

**Figure 4:**
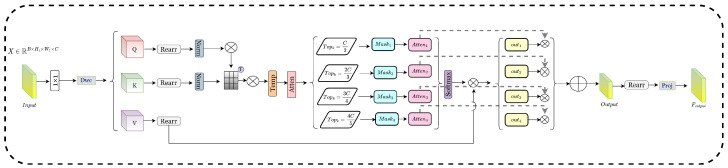
Top-k dynamic mask (TDM).

**Table 1:** Quantitative comparison with the recent SOTA methods on three benchmark datasets. ↑/↓ denotes that larger/smaller is better. “-” is not available. The top two models are **bolded** and underlined, respectively. Red and blue indicate the best and second performance respectively. Same explanations apply to the following tables.

Method	Year	Backbone	CAMO (250)					COD10K (2026)					NC4K (4121)				
			$S_{m}↑$	$F_{\beta }^{\omega }↑$	M↓	$F_{\beta }^{m}↑$	$E_{\phi }^{m}↑$	$S_{m}↑$	$F_{\beta }^{\omega }↑$	M↓	$F_{\beta }^{m}↑$	$E_{\phi }^{m}↑$	$S_{m}↑$	$F_{\beta }^{\omega }↑$	M↓	$F_{\beta }^{m}↑$	$E_{\phi }^{m}↑$
ResNet50-based Methods																	
BGNet [3]	22IJCAI	ResNet	0.812	0.749	0.073	0.789	0.870	0.831	0.722	0.033	0.753	0.901	0.851	0.788	0.044	0.820	0.907
BSANet [36]	22AAAI	ResNet	0.794	0.717	0.079	0.763	0.851	0.818	0.699	0.034	0.738	0.891	0.842	0.771	0.048	0.808	0.897
SegMaR [37]	22CVPR	ResNet	0.816	0.753	0.071	0.795	0.874	0.833	0.724	0.034	0.757	0.899	0.841	0.781	0.046	0.821	0.896
ZoomNet [35]	22CVPR	ResNet	**0.820**	0.752	**0.066**	0.793	0.877	**0.838**	0.729	**0.029**	0.766	0.888	0.853	0.784	0.043	0.818	0.896
C2FNetV2 [2]	22TCSVT	ResNet	0.799	0.730	0.077	0.770	0.859	0.811	0.691	0.036	0.725	0.887	0.840	0.770	0.048	0.802	0.896
FEDER [38]	23CVPR	ResNet	0.802	0.738	0.071	0.781	0.867	0.822	0.716	0.032	0.751	0.900	0.847	0.789	0.044	0.824	0.907
ICEG [39]	24ICLR	ResNet	0.810	0.727	0.068	0.789	0.879	0.826	0.698	0.030	0.747	0.906	0.849	0.760	0.044	0.814	0.908
CamoFormer [19]	24ICLR	ResNet	0.817	0.752	0.067	0.792	0.866	0.838	0.723	0.029	0.753	0.906	0.849	0.760	0.044	0.814	0.908
**ATDM-R50**	Ours	ResNet	0.815	**0.754**	**0.068**	**0.797**	**0.885**	**0.835**	**0.742**	**0.029**	**0.773**	**0.910**	**0.854**	**0.800**	**0.041**	**0.832**	**0.915**
Res2Net50-based Methods																	
FDNet [40]	22CVPR	Res2Net	0.838	0.774	0.063	0.806	0.897	0.834	0.727	0.030	0.753	**0.921**	0.828	0.748	0.052	0.780	0.894
SINetV2 [1]	22TPAMI	Res2Net	0.820	0.743	0.071	0.782	0.882	0.815	0.680	0.037	0.718	0.887	0.847	0.770	0.048	0.805	0.903
DINet [32]	24TMM	Res2Net	0.821	0.748	0.068	0.790	0.873	0.832	0.724	0.031	0.761	0.903	0.856	0.790	0.043	0.825	0.909
FCOCM [33]	24TCSVT	Res2Net	0.836	0.781	0.063	0.815	0.893	0.837	0.726	0.030	0.755	0.903	0.860	0.799	**0.041**	0.827	0.913
**ATDM-R2N**	Ours	Res2Net	**0.840**	**0.787**	**0.063**	**0.820**	**0.899**	**0.845**	**0.743**	**0.028**	**0.775**	0.912	**0.861**	**0.806**	**0.042**	**0.835**	**0.915**
Transformer-based Methods																	
FPNet [41]	23ACMMM	PVT	0.851	0.802	0.056	0.836	0.905	0.850	0.755	0.028	0.782	0.912	-	-	-	-	-
FSPNet [42]	23CVPR	VIT	0.856	0.799	0.050	0.830	0.899	0.851	0.735	0.026	0.769	0.895	0.879	0.816	0.035	0.843	0.915
SARNet [34]	23TCSVT	PVT	0.868	0.828	0.047	0.850	0.927	0.864	0.777	0.024	0.800	0.931	0.886	0.842	0.032	0.863	0.937
UEDG [4]	23TMM	PVT	0.863	0.817	0.048	0.840	0.922	0.858	0.766	0.025	0.791	0.924	0.879	0.830	0.035	0.851	0.929
VSCode [43]	24CVPR	Swin	0.873	0.820	0.046	0.844	0.925	0.869	0.780	0.023	0.806	0.931	0.891	0.841	0.032	0.863	0.935
RISNet [44]	24CVPR	PVT	0.870	0.827	0.050	0.844	0.922	0.873	0.799	0.025	0.817	0.931	0.882	0.834	0.037	0.854	0.926
PRNet [5]	24TCSVT	SMT	0.872	0.831	0.050	0.855	0.922	0.874	0.799	0.023	0.822	0.937	0.891	0.848	0.031	0.869	0.935
**ATDM-PVT**	Ours	PVT	**0.882**	**0.846**	0.044	**0.867**	**0.933**	**0.879**	**0.804**	**0.021**	**0.823**	**0.940**	**0.895**	**0.852**	**0.030**	**0.872**	**0.940**
**ATDM-Swin**	Ours	Swin	0.879	0.836	**0.041**	0.858	0.932	0.868	0.781	0.023	0.803	0.934	0.891	0.845	0.031	0.864	**0.940**

**Table 2:** Computational efficiency comparison of SOTA COD methods.

Method	Backbone	Params (M)	MACs (G)	Speed (FPS)
BGNet [3]	ResNet50	26.5	45.2	62.1 ± 1.2
ZoomNet [35]	ResNet50	45.7	68.7	58.3 ± 1.5
CamoFormer [19]	ResNet50	38.2	53.9	74.5 ± 1.1
**ATDM-RN (Ours)**	ResNet50	**28.3**	**47.8**	**68.2 ± 1.2**
FDNet [40]	Res2Net50	28.7	45.6	85.6 ± 2.1
DINet [40]	Res2Net50	45.2	62.5	72.5± 1.6
SINetV2 [40]	Res2Net50	51.1	68.5	65.3 ± 2.6
**ATDM-R2N (Ours)**	Res2Net50	**29.5**	**43.2**	**88.4 ± 1.3**
FPNet [41]	PVT	68.4	89.2	45.3 ± 3.4
SARNet [34]	PVT	52.1	89.4	38.2 ± 3.4
**ATDM-PVT (Ours)**	PVT	**36.8**	**62.1**	**89.5 ± 1.8**
PRNet [5]	SMT	34.6	76.8	45.1 ± 1.7
**ATDM-Swin (Ours)**	Swin-T	**27.2**	**58.6**	**92.5 ± 2.3**

**Table 3:** Ablation study results (performance comparison on CAMO, COD10K, and NC4K datasets).

Model Configuration	CAMO			COD10K			NC4K		
	$F_{\beta }↑$	$E_{\phi }↑$	M↓	$F_{\beta }↑$	$E_{\phi }↑$	M↓	$F_{\beta }↑$	$E_{\phi }↑$	M↓
Baseline (Res2Net)	0.721	0.862	0.075	0.742	0.887	0.032	0.738	0.890	0.045
Baseline + MHA	0.748	0.880	0.068	0.769	0.903	0.029	0.761	0.902	0.041
Baseline + TDM	0.743	0.875	0.070	0.763	0.896	0.030	0.756	0.899	0.042
ATDMNet	0.765	0.895	0.063	0.785	0.915	0.025	0.778	0.778	0.038

**Table 4:** Performance with varying quantities of agent nodes.

Agent Nodes	COD10K				CAMO				NC4K			
	$F_{\beta }↑$	M↓	$E_{\phi }↑$	$S_{\alpha }↑$	$F_{\beta }↑$	M↓	$E_{\phi }↑$	$S_{\alpha }↑$	$F_{\beta }↑$	M↓	$E_{\phi }↑$	$S_{\alpha }↑$
4	0.72	0.12	0.68	0.75	0.69	0.14	0.65	0.70	0.68	0.13	0.67	0.72
8	0.78	0.10	0.72	0.78	0.75	0.12	0.69	0.73	0.73	0.11	0.71	0.76
16	0.83	0.08	0.76	0.82	0.81	0.09	0.74	0.78	0.79	0.09	0.69	0.79
32	0.81	0.09	0.74	0.80	0.79	0.10	0.72	0.76	0.77	0.10	0.70	0.75
64	0.79	0.11	0.71	0.79	0.77	0.11	0.70	0.75	0.75	0.12	0.68	0.73

**Table 5:** Performance across various top-k ratios.

Top-k Ratios	COD10K				CAMO				NC4K			
	$F_{\beta }↑$	M↓	$E_{\phi }↑$	$S_{\alpha }↑$	$F_{\beta }↑$	M↓	$E_{\phi }↑$	$S_{\alpha }↑$	$F_{\beta }↑$	M↓	$E_{\phi }↑$	$S_{\alpha }↑$
50%	0.78	0.10	0.72	0.78	0.73	0.11	0.68	0.74	0.71	0.12	0.66	0.70
60%	0.81	0.09	0.75	0.80	0.78	0.10	0.71	0.76	0.75	0.10	0.69	0.73
70%	0.84	0.07	0.78	0.83	0.80	0.08	0.74	0.79	0.79	0.08	0.72	0.77
80%	0.81	0.08	0.76	0.81	0.81	0.09	0.73	0.77	0.77	0.09	0.70	0.75
90%	0.80	0.09	0.74	0.80	0.79	0.10	0.71	0.76	0.76	0.10	0.69	0.74

## Data Availability

The datasets produced and examined and the ATDMNet source code in this study are now freely accessible on GitHub at https://github.com/dfryeel/free.git (accessed on 1 March 2025). This study did not generate any new datasets. For further information or materials, please reach out to the corresponding author.
